# A *Streptococcus mutans* retrospective: from oral pathogen to bacterial paradigm

**DOI:** 10.1128/jb.00555-25

**Published:** 2026-02-13

**Authors:** Lin Zeng, Jacqueline Abranches, José A. Lemos

**Affiliations:** 1 Department of Oral Biology, University of Florida College of Dentistry 164889, Gainesville, Florida, USA; Dartmouth College Geisel School of Medicine, Hanover, New Hampshire, USA

**Keywords:** microbial interactions, sugar metabolism, genetic competence, biofilm, gram-positive model organism, *Streptococcus mutans*, model organism

## Abstract

From its discovery in the late 1800s to the present day, *Streptococcus mutans* research has been at the forefront of dental caries investigations, providing the foundation for our current understanding of the microbiological determinants of this disease. In addition, research on *S. mutans* has greatly advanced key areas of microbiology, establishing the organism as a notably useful model for understanding the biology of other streptococcal species, as well as Gram-positive bacteria more broadly. In this piece, we provide a retrospective of how *S. mutans* has served this dual role, highlighting specific areas that have benefited most from *S. mutans*–driven discoveries. We close by outlining emerging areas of *S. mutans* research that are likely to influence future breakthroughs in both oral health science and basic microbiology.

## THE RISE OF *STREPTOCOCCUS MUTANS* IN CARIES RESEARCH

**Fig 1:**
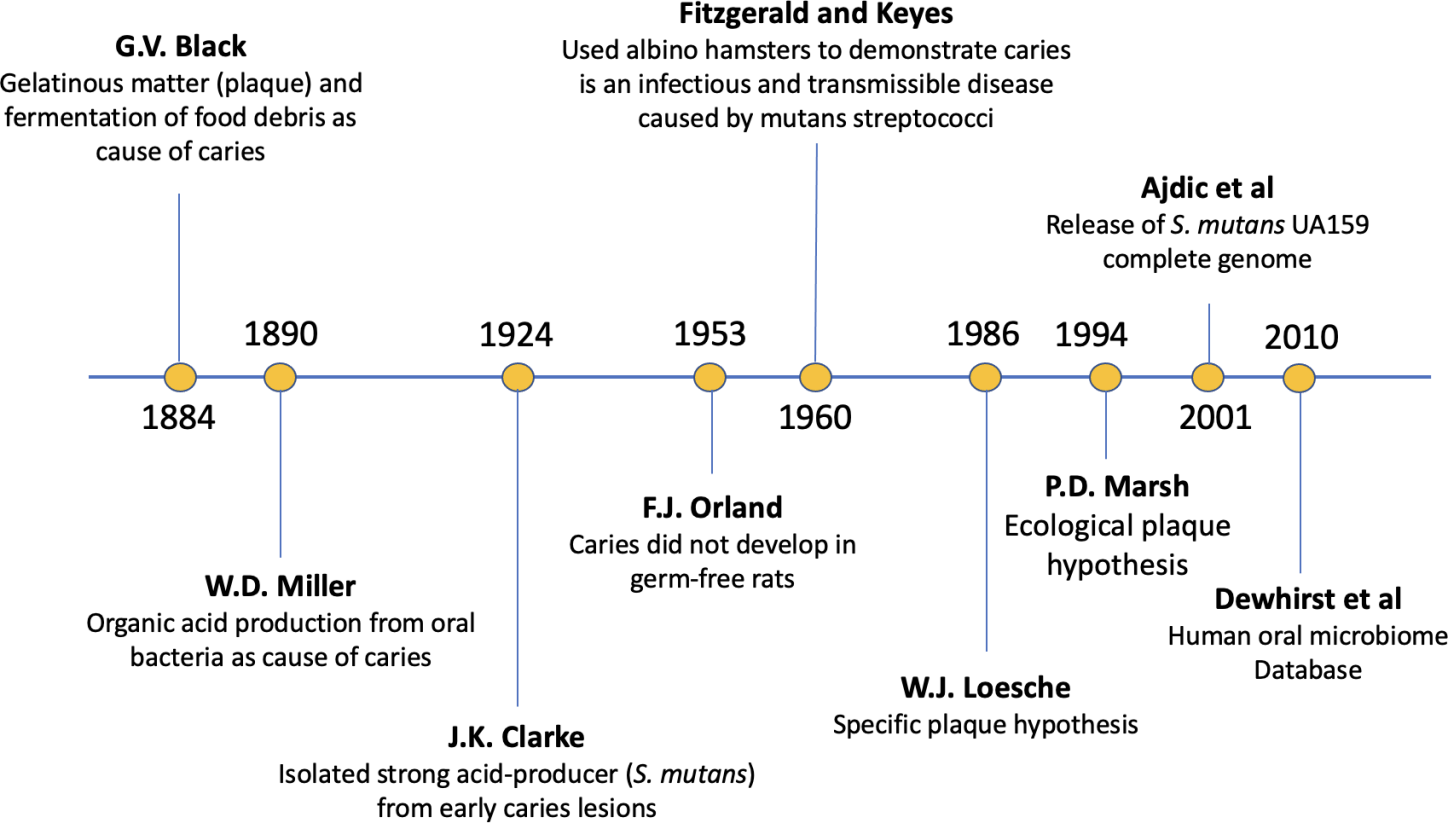
Timeline of *S. mutans* research milestones.

From the ancient “tooth worm” theory to the modern understanding of dental caries as a multifactorial disease driven by complex microbial interactions and influenced by host factors such as saliva composition and flow, carbohydrate intake, oral hygiene, and fluoride exposure, the history of *Streptococcus mutans* is deeply intertwined with the history of dental caries research (Fig. 1). Building on Pasteur’s work describing the requirement of microorganisms in fermentation and Koch’s Germ Theory, studies published around the late 1800s pioneered the idea that dental caries resulted from the corrosive action of organic acids produced by bacteria present in the mouth. Without discounting contributions of many other researchers during this era—a comprehensive timeline of the theories and research conducted during this time can be found here (1); it was the work of W. D. Miller (2) showing demineralization of extracted teeth soaked in saliva, bread (as a food source), and bacteria that bolstered the microbially-driven nature of the disease dental caries. Furthermore, it was also around this time that researchers began associating the sticky gelatinous matter stuck to teeth, known as dental plaque, with carious lesions. However, it was only in 1924 when J. K. Clarke isolated from carious lesions a strong acid-producing bacterium that was capable of demineralizing tooth sections *ex vivo* that the first specific microbial culprit of dental caries was officially identified; he named this bacterium *S. mutans* due to its irregularly shaped appearance under the microscope (3). The infectious and transmissible nature of dental caries was confirmed about 30 years later. First, F. J. Orland et al. showed that carious lesions did not develop in germ-free rats (4), which was closely followed by the work of R. J. Fitzgerald and P. Keyes demonstrating that oral infection with a pure culture of an oral *Streptococcus* strain, later identified as *S. cricetus*, could be transmitted from dams to pups and cause caries in albino hamsters (5). From subsequent studies, it became clear that only a relatively small subset of acidogenic oral bacteria could initiate caries lesions in animal models, with the majority assigned to members of the mutans streptococci group, including *S. mutans*, *S. cricetus*, *S. ratti* (formerly *S. rattus*), and *S. sobrinus* (6). It was also around this period that experimental evidence establishing that a high-sucrose diet and the capacity of mutans streptococci to synthesize extracellular polysaccharides of glucan was obtained (more on this below). Since then, our understanding of the microbiological aspects of dental caries has continued to evolve, from the Specific Plaque Hypothesis, which identified mutans streptococci (particularly *S. mutans*) and lactobacilli as key etiological agents of dental caries (6), to the Ecological Plaque Hypothesis proposing that environmental stresses trigger a shift in the oral microbial community that results in the enrichment of cariogenic microorganisms (7), to more recent insights informed by contemporary oral microbiome and metagenomic studies that came on the footsteps of next-generation sequencing technology. While generally supportive of the central role of *S. mutans* in caries development, these studies have also reinforced the polymicrobial nature of caries. A comprehensive perspective of research in this area can be found in reference 8.

## STRESS RESPONSES, BIOFILM LIFESTYLE, AND THE EMERGENCE OF A NEW BACTERIAL PARADIGM

With the advancements of biochemical, serological, and molecular technologies for bacterial identification, it became clearer that *S. mutans* is the predominant species of mutans streptococci associated with dental caries in humans. Armed with this understanding, from the 1980s onward, studies aimed at uncovering factors that contribute to the cariogenicity of *S. mutans* surged. This research boom was greatly facilitated by the discovery that *S. mutans* is genetically competent (9) and therefore very amenable to genetic manipulations, and the release of the first complete *S. mutans* genome sequence, the serotype *c* strain UA159 (10). Today, it is recognized that the cariogenic potential of *S. mutans* stems from three key attributes: (i) ability to produce large quantities of extracellular glucan polymers from sucrose, (ii) ability to transport and ferment a wide range of dietary carbohydrates, and (iii) ability to thrive in low pH environments while also tolerating various other environmental stresses. Regarding the latter, it was between the 1990s and early 2000s that critical aspects of the adaptive acid stress response of *S. mutans*, characterized by an increased resistance to acid killing in cells grown at a non-lethal acidic pH, were uncovered. Among them, it was found that acid tolerance of oral streptococci correlates with the pH optimum of the membrane-bound F-ATPase, an H^+^-translocating pump that functions optimally at pH 6 in *S. mutans* but at pH 7 or above in less acid-tolerant species such as *S. salivarius* and *S. sanguinis* (11). Moreover, as the environmental pH becomes more acidic, the composition of the plasma membrane of *S. mutans* was found to rapidly shift from primarily short-chain saturated fatty acids to long-chain monounsaturated fatty acids (12). This flip in the membrane fatty acid profile was shown to contribute to acid tolerance by altering membrane permeability to H^+^ ions and through modulation of F-ATPase activity (13).

**Fig 2:**
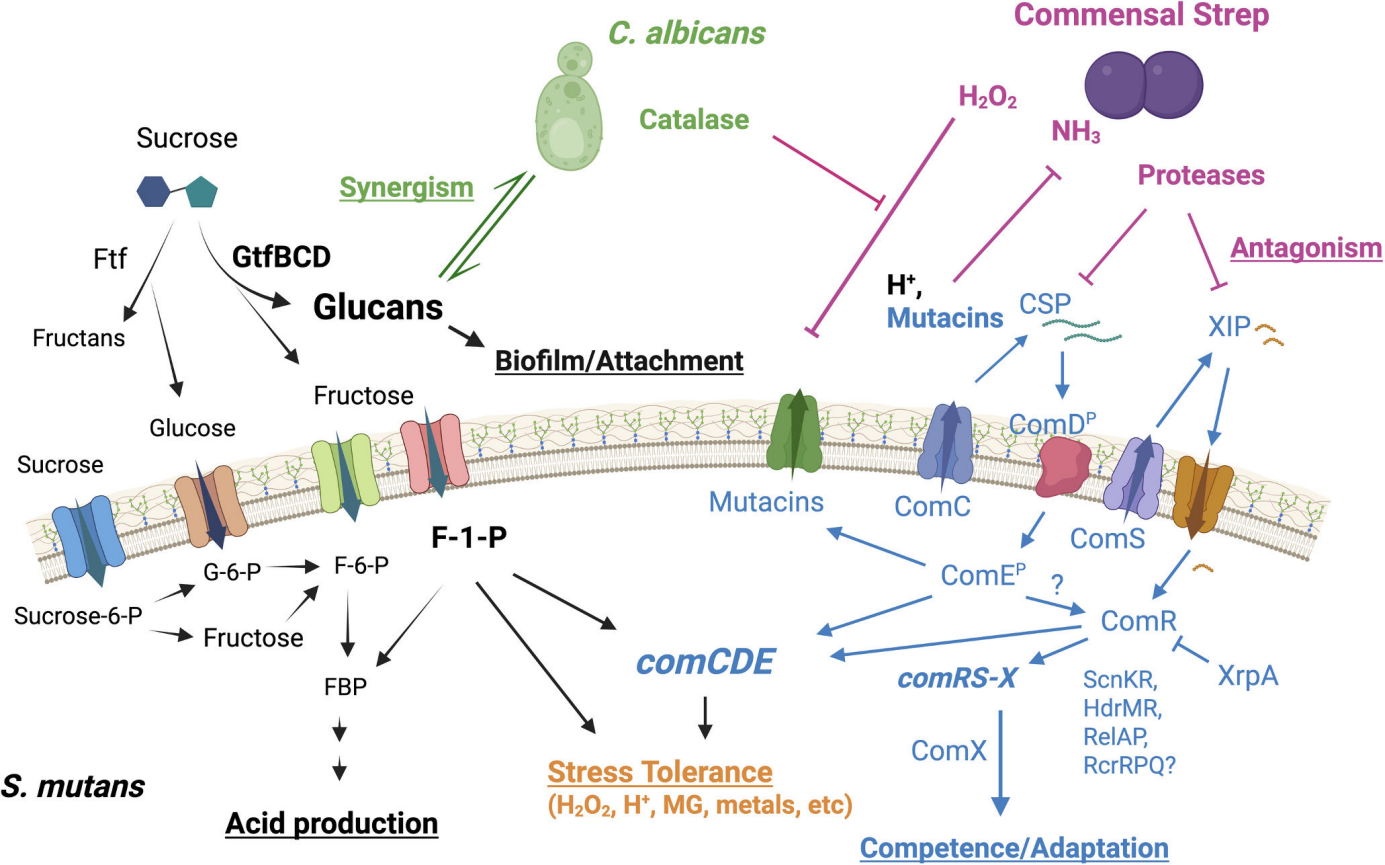
Carbohydrate metabolism, stress tolerance, and quorum-sensing signaling pathways are intertwined and contribute to *S. mutans’* competitive fitness within oral biofilms.

With the appreciation that most bacterial infections have a biofilm nature and the genomic revolution in the early 2000s, *S. mutans* research gained a new and broader dimension. Specifically, the organism’s host-associated biofilm lifestyle, genetic tractability, and easy access to fresh clinical samples placed *S. mutans* in a position to not only advance dental research but also to serve as a model Gram-positive organism (14). For example, a great breadth of knowledge was obtained from studies focused on understanding the developmental stages and complex inter-microbial interactions within the biofilm space. Also, these studies revealed how obligately host-associated bacteria cope with stresses and integrate their stress regulon with other biological functions, such as biofilm formation, carbohydrate metabolism, and genetic competence (Fig. 2) (15, 16). Rather than trying to cover all aspects of *S. mutans* research, in the sections below, we will highlight three specific areas, namely biofilm development, carbohydrate metabolism, and genetic competence (Fig. 2), that have broadly benefited from *S. mutans* investigations.

## *S. MUTANS* GLUCAN MATRIX: A BIOFILM FORMATION PARADIGM

One of the most recognized features of *S. mutans* is its ability to secrete enzymes called glucosyltransferase (GTFs) that convert sucrose into extracellular glucans—homopolymers of glucose found in water-soluble and water-insoluble forms—that facilitate the permanent colonization of hard tooth surfaces by promoting the development of highly adhesive and cohesive biofilms. The earliest report on this type of activity dates back to 1886 with the description of a sticky, gelatine-like substance produced by streptococci grown in sucrose-containing medium (17). These water-soluble glucans are primarily constructed by α-1,6 bonds and degraded by dextranases, potentially serving as a temporary energy reserve. On the other hand, water-insoluble glucans are rich in α-1,3 linkages and, through the activity of several glucan-binding proteins expressed at the bacterial surface, are primarily responsible for *S. mutans’* remarkable ability to form biofilms in the presence of sucrose. GTF enzymes are present in most *S. mutans* isolates and several other oral streptococci, with the genome of *S. mutans* UA159 encoding three well-characterized isoenzymes that catalyze the formation of water-soluble (GtfD), water-insoluble (GtfB), or a mixture of soluble and insoluble (GtfC) glucans. Although the structure and function of glucans and the roles of individual GTFs in biofilm formation are of great importance and have been thoroughly investigated, these topics fall outside the scope of this article. Readers seeking further information on *S. mutans* biofilms and GTFs are encouraged to consult the following reviews (18–20).

Of interest here are discoveries that pertain to biofilm dynamics and contributions of the GTFs to the recruitment of other caries-associated microorganisms to the biofilm matrix. In earlier studies, secreted GtfB was shown to bind to other oral bacteria, turning GTF-negative bacteria into *de facto* glucan producers (21). As many oral microbes lack the ability to adhere to tooth surfaces, the unique properties of surface-adsorbed GTFs were shown to promote microbial aggregation and adherence to dental pellicles, allowing for efficient biofilm development, which is a prerequisite for caries initiation. This notion was later expanded and exemplified by the identification of synergistic cross-kingdom interactions between *S. mutans* and the fungal pathobiont *Candida albicans,* which are linked to higher caries severity (more on the *S. mutans–C. albicans* cross-kingdom interaction below). Several technological advancements for studying oral biofilms, both *in vitro* and *ex vivo*, have reinforced the pivotal role of *S. mutans* GTFs in the initiation and progression of dental caries by driving the formation of a highly adherent, diffusion-limiting, proton-trapping (low pH) matrix that is conducive to both demineralization of the tooth structure and eventual selection of an aciduric (cariogenic) microbiota. These studies have significantly advanced biofilm research by establishing foundational knowledge on the spatial distribution, interspecies interactions, and physicochemical properties of complex biofilms.

## CARBOHYDRATE METABOLISM: THE SWEET SECRETS OF *S. MUTANS*

Aside from the utilization of sucrose for glucan synthesis, the ability of *S. mutans* to import and ferment a large array of carbohydrates is also viewed as a critical trait in its pathophysiology. In fact, an important aspect of *S. mutans* cariogenicity is that most isolates can continue to carry out glycolysis under acidic conditions, as low as pH 4.4, driving the process of acidification and microbial dysbiosis in dental plaque further (22–24). Due to this association, research on carbohydrate metabolism by *S. mutans* had a head start relative to other streptococci. Earlier investigations focused on characterizing genetic factors required for the transport and catabolism of carbohydrates abundant in dental biofilms, including sucrose (25), lactose (26), and complex carbohydrates derived from sucrose, including fructans, a fructose polymer, and glucans (27). Similar investigations were later performed on less common carbohydrates, such as galactose (28), cellobiose (29), the amino sugars glucosamine and N-acetylglucosamine (30), trehalose (31), and raffinose (32). Collectively, these studies revealed that *S. mutans* possesses a high number of sugar-specific phosphotransferase systems (PTS) that are primarily responsible for carbohydrate uptake (33, 34).

As the reader should appreciate in other parts of this text, *S. mutans* has not only served as a model organism for novel discoveries, but research in the field has also challenged long-standing dogmas derived from studies of traditional bacterial models such as *Escherichia coli* and, in particular, the Gram-positive paradigm *Bacillus subtilis*. For example, genetic analysis of carbohydrate catabolic systems soon identified several noteworthy phenotypes for *S. mutans* in prioritization of carbohydrate catabolism, in particular those related to the role of the major catabolite control protein CcpA, and the glucose-PTS. Specifically, these investigations uncovered the central role of the glucose-PTS (29, 35, 36), the absence of strong phenotypes in the *ccpA* mutant (37), and the discovery that sugar concentrations required for regulation by the PTS are lower than those needed for CcpA-dependent control (38). Ultimately, these studies revealed a PTS-centric model of carbon catabolite regulation in *S. mutans*, a departure from the CcpA-centered paradigm established in *B. subtilis* (39, 40). Moreover, subsequent studies returned similar findings regarding the central role of the PTS in regulating catabolic activities in commensal streptococci and in coupling the regulation of carbohydrate metabolism with virulence expression in pathogenic streptococci (41–43).

In recent years, the negative impact of overconsumption of fructose on human health has gained increased public awareness. An important recent development is the appreciation of the potential influence of fructose metabolism on *S. mutans* pathophysiology. Previous studies have shown that *S. mutans* harbors at least three fructose PTS transporters, FruI, FruCD, and LevD, and all enzymes needed to metabolize fructose through both fructose-1-phosphate (F-1-P) and fructose-6-phosphate (F-6-P) pathways (44, 45). Additionally, *S. mutans* encodes an F-1-P-specific phosphohydrolase, SppA, that modulates intracellular F-1-P levels (46). An earlier animal study indicated that the *S. mutans* F-1-P-generating PTS, the FruRKI pathway, was required for oral colonization and induction of dentin caries in rats fed a high-sucrose diet (47). Recent research has begun to uncover the importance of fructose metabolism, through F-1-P in particular, in streptococcal fitness and stress tolerance (Fig. 2). Specifically, elevation of intracellular F-1-P, through treatment with fructose or deletion of either SppA or the F-1-P kinase FruK, led to stress-like phenotypes, such as growth arrest, competence, autolysis, and enhanced biofilm accumulation (46, 48, 49). In addition, fructose significantly impacted *S. mutans* physiology, with the UA159 strain displaying an extended lag phase before transitioning into growth on a different carbohydrate source such as lactose (50). This memory-like effect of fructose, and associated cheating behavior in a heterogeneous population, was later shown to enhance the bacterium’s competitiveness when faced with shifting carbohydrate sources (51) and, in the case of lactose, was partly due to the ability of F-1-P to interact with the lactose regulator LacR, which inhibits expression of the *lac* operon (52). Finally, recent studies reported phenotypes and revealed the underlying mechanisms for fructose-induced stress response and enhanced tolerance under low pH, starvation, and oxidative stress conditions (53). A comparative RNA-Seq analysis identified a common stress core of more than 60 genes that is affected by fructose, methylglyoxal, a reactive electrophile species generated during glycolysis (54), and hydrogen peroxide. In several other streptococci, the orthologous F-1-P metabolic operon (*fruRBA/fruRKI*) has been associated with overall fitness, biofilm development, and virulence (55–58). Given the presence of fructose in both the oral cavity and the bloodstream (59), these new developments are likely relevant to other important human pathogens.

## COMPETENCE AND STRESS: THE TANGLED SIGNALING WEB OF *S. MUTANS*

Another important reason that made *S. mutans* the laboratory workhorse it is today was the early discovery that the species can enter a genetic competent state under *in vitro* conditions (9). In principle, natural competence transiently allows uptake and integration of exogenous DNA into the chromosome of the competent organism, enhancing genetic diversity and survivability when faced with adverse environmental conditions. In *S. mutans*, activation of the competence state is manifested as a complex network comprised of at least two main regulatory systems (Fig. 2), ComCDE and ComRS-ComX, both of which require the release and detection of small peptide pheromones, with competence-stimulating peptide (CSP) derived from ComC (60) and ComX-inducing peptide (XIP) derived from ComS (61). This differs from two well-characterized competence systems, *S. pneumoniae* (62) and *B. subtilis* (63)*,* which are each controlled by one main regulatory system. The ComRS-ComX pathway is the proximal circuit in *S. mutans*, consisting of the Rgg-like transcription regulator ComR that responds to intracellular XIP signals and downstream alternative sigma factor ComX that directly induces expression of the late competence genes to mediate DNA uptake and homologous recombination (62). ComRS is essential for competence development in *S. mutans,* yet ComDE is not. The ComRS-ComX system is also present in Pyogenic, Bovis, and Salivarius groups of streptococci and appears similarly indispensable for their competence development (62). Although the association of the two-component system ComDE and CSP pheromone with *S. mutans* competence precedes the identification of ComRS, it is now recognized that the system is primarily responsible for controlling the production of bacteriocins, known as mutacins, and its association with competence development, which includes a feedback loop between ComRS and ComCDE, remains to be fully elucidated (64, 65).

Consistent with its biofilm-specific lifestyle, research in the last decades has also revealed that *S. mutans* integrates stress response and quorum sensing at both the individual cell and population levels, with some suggesting that the streptococcal ComCDE system represents a general stress response mechanism that resembles the canonical SOS response of *E. coli* (66). In *S. mutans* UA159, stressors such as low pH, H_2_O_2_, heat, antibiotics, and amino acid starvation have all been shown to induce the ComCDE pathway (67, 68), which mediates killing of a fraction of the population and the formation of persister cells in another part of the population (67, 69). This type of sub-population behavior is a result of stochastic or bimodal response and was later characterized in detail for CSP-mediated competence development (70), a phenotype attributed to the positive feedback loop of the ComRS-ComX circuit that cross-activates the ComCDE system (71, 72). As a result, through activation of the ComCDE pathway, CSP induces a uniform population response to produce mutacins that target sensitive competitors (65). The exact mechanisms for this differential response to CSP between two related phenotypes remain to be clarified; however, the biological outcome is that mutacin production is activated in a cell density-dependent manner (true quorum sensing), whereas competence development is sensitive to modulation by environmental factors. In contrast, CSP in *S. pneumoniae* induces a synchronized competence window (62), whereas *B. subtilis* (63) presents a stochastic competence like *S. mutans* that is regulated by factors such as stress and media compositions (70). While ComCDE mostly controls the expression of a subset of bacteriocins called non-lantibiotic mutacins that are effective at killing related streptococci, a third quorum-sensing system, MutRS, was recently identified in *S. mutans* (73) and shown to control several lantibiotic mutacins that target a wide spectrum of Gram-positive bacteria (65).

The complex nature of competence development in *S. mutans* and its integration with stress response pathways has been further supported by studies describing the Rel competence-related (RcrRPQ) pathway, which is comprised of a MarR-type transcription regulator, RcrR, and a pair of ABC transporters, RcrP and RcrQ (74). Compared to the ComCDE and ComRS-ComX circuits, RcrRPQ is even more enigmatic in its influence on cell stress physiology and competence development. First, two small peptides (27 and 42 aa) were identified near the 3′-end of the *rcrQ* coding sequence and shown to modulate both competence and sensitivity to CSP-induced cell death (75). This discovery led to the eventual identification of a ComR-binding inhibitor of competence, XrpA, which itself is another cryptic small protein (69 aa) encoded within the *comX* gene (76). Furthermore, *rcrRPQ* is adjacent to *relP*, encoding an enzymatic source of the stringent response effector (p)ppGpp (77). By interrogating the interplay between (p)ppGpp metabolism and XIP-dependent death and competence phenotype, the authors proposed that (p)ppGpp homeostasis, in concert with optimal signals from quorum sensing and several stress-responsive circuits, dictates the percentage of the bacterial population undergoing transformation or death (78). It is worth noting that despite significant divergence in competence and underlying genetics within the *Streptococcus* genus (79), the *rcrRPQ* cluster is well conserved among streptococci, excluding *S. pneumoniae,* and shown to contribute to stress tolerance in the oral commensal *S. gordonii* (80). Further research may clarify whether the integrated regulation of competence and stress tolerance is unique to *S. mutans* as an oral pathobiont or represents a conserved trait among other species within the genus.

Today, our understanding of the genetic mechanisms that control *S. mutans* competence development has surpassed *S. pneumoniae*—the first naturally competent organism described in the literature—revealing an extremely complex network that includes several additional regulators that modulate ComX activity, including RcrR (74), HdrRM (81), and ScnKR (82). An in-depth discussion of this topic can be found in the review by Kaspar and Walker (83).

## ORAL BIOFILMS AS A MODEL FOR MICROBIAL COOPERATION AND COMPETITION

In addition to advancing our understanding of the role of the extracellular matrix in biofilm initiation, development, and maturation, research focused on *S. mutans’* relationship with specific members of the supragingival biofilm (dental plaque) community has yielded important insights into how interspecies, and even interkingdom, interactions shape biofilm ecology and influence the balance between oral health and disease. These interactions can be competitive/antagonistic, as in the case of commensal streptococci of the Mitis group, or have a mutualistic/synergistic nature as exemplified by the pathobiont yeast *C. albicans*. Below, we briefly discuss these specific examples.

## ANTAGONISTIC INTERACTIONS

Occupying the same ecological niche, commensal streptococci, such as *S. sanguinis* and *S. gordonii*, along with *S. mutans*, share many physiological features, including the absence of a complete Krebs (tricarboxylic acid) cycle and a reliance on carbohydrate fermentation as their primary means of ATP generation (84, 85). From the perspective of *S. mutans*, the production of mutacins and its capacity to adapt and survive in acidic environments lethal to most oral streptococci are considered key factors in its competitive fitness against commensal streptococci, especially under high sucrose conditions (65).

Despite encoding GTF enzymes (86, 87), members of the Mitis group are not known for a capacity to promote robust biofilm formation via extracellular glucan production. On the other hand, members of this group possess a number of specialized adhesins that mediate attachment to host-derived proteins present on salivary pellicles, thereby functioning as pioneer colonizers of the supragingival biofilm (88–91). Over the years, numerous studies have linked *S. sanguinis* abundance in dental plaque with oral health (92–95), with clinical studies reporting an inverse correlation between the abundance of commensal streptococci and mutans streptococci/*S. mutans* in healthy plaque (high Mitis streptococci/low mutans streptococci) and carious lesions (low Mitis streptococci/high mutans streptococci) (95–99). Mechanistically, *S. sanguinis*, *S. gordonii,* and other health-associated streptococci are net producers of H_2_O_2_ that is inhibitory to *S. mutans*, with some species capable of modulating the environmental pH via ammonia-generating pathways, such as urease and the arginine deiminase system (100). In peroxigenic streptococci, H_2_O_2_ production can be mediated by pyruvate oxidase (SpxB or Pox) and lactate oxidase (Lox) enzymes (101, 102) and at least one amino acid oxidation pathway (103, 104). Recently, glycerol metabolism via the phosphorylation pathway GlpKOF, conserved in many streptococci but not in *S. mutans*, was shown to be another source for H_2_O_2_ production and to contribute to the competitive fitness of *S. sanguinis* against *S. mutans* (105). In addition, catabolism of the amino sugar GlcNAc was shown to enhance the competitiveness of commensal streptococci against *S. mutans* through the release of ammonia, with the added benefit of shunting pyruvate from production of lactic acid to the weaker acetic acid in a process that generates H_2_O_2_ and an extra ATP molecule (106, 107). A less explored mechanism utilized by commensal streptococci to compete in the supragingival biofilm is through the production of enzymes that disrupt activation of *S. mutans* quorum-sensing systems. For example, *S. gordonii* strains encode Challisin (Sgc) (108), a serine protease that degrades CSP, disrupting activation of ComCDE, which is necessary for mutacin production (65). Notably, Sgc-like serine proteases have been described in *S. sanguinis* (109) and in *Streptococcus* sp. A12, an isolate from a caries-free individual with probiotic potential due to possessing multiple anti-cariogenic properties that included an endopeptidase, PcfO, that blocks the XIP competence signaling pathway (110, 111).

## SYNERGISTIC INTERACTIONS

The yeast *C. albicans* is the most common species associated with humans and an important opportunistic pathogen associated with mucosal, skin, and systemic infections (112). In addition, several studies have shown that *C. albicans* is enriched in the dental plaque of caries-active subjects, where it can be found associated with *S. mutans* and other caries-associated bacteria, such as *Scardovia wigsiae*, *Lactobacillus* spp*.,* and *Veillonella* (113–115). From a clinical standpoint, co-infection with *S. mutans* and *C. albicans* has been strongly associated with early childhood caries (116–122), and microscopic analysis has revealed *S. mutans* microcolonies physically associated with *C. albicans* near dental enamel areas where demineralization can be observed (123).

Despite possessing a number of surface adhesins, evidence suggests that *C. albicans* relies on co-adhesion with oral streptococci to colonize and persist in the oral cavity (124, 125). In addition, there are several reasons indicating that *C. albicans* and oral streptococci benefit from each other’s presence. For example, *C. albicans* can utilize lactic acid as an energy source, the major fermentation end product secreted by all streptococci, while also secreting a number of bacterial/biofilm growth factors and reducing oxygen tension of oral biofilms (124, 125). In co-culture, *C. albicans* increases *S. mutans* carbohydrate metabolism and acid tolerance, traits that are essential for caries development (126–130). In a clear demonstration of the synergistic interactions between *C. albicans* and *S. mutans*, *in vitro* biofilms containing both species displayed increased biomass and heightened tolerance to environmental challenges, such as chlorhexidine, heat, acid, and osmotic stresses, when compared to individual single-species biofilms (131, 132). Mechanistically, the *S. mutans–C. albicans* co-culture enhances glucan production as *S. mutans*-secreted GtfB binds to fungal cell wall mannans, converting *C. albicans* into a *de facto* α-glucan producer (*C. albicans* can synthesize β-glucan as part of its cell wall) in the presence of sucrose while also stimulating expression of *C. albicans*-specific genes involved in biofilm development (133–135). This association has been further supported by studies showing that purified GtfB or *S. mutans* membrane vesicles containing GTFs as cargo stimulate biofilm formation (136–139). Finally, *C. albicans* encodes a catalase enzyme shown to cross-protect *S. mutans* against peroxide stresses, including H_2_O_2_ secreted by peroxigenic oral streptococci (140). It was proposed that *S. mutans–C. albicans* assemble as a cross-kingdom “supraorganism” with increased fitness that can rapidly grow and spread on surfaces, resulting in increased caries risk (141). The clinical association of *S. mutans* and *C. albicans* in severe caries and the *in vitro* evidence obtained so far is supported by rat caries studies showing that co-infection with *C. albicans* and *S. mutans* leads to higher carriage of both microorganisms in dental plaque and more rapid and aggressive caries progression (134, 142). Interestingly, in advanced dentin lesions, *S. mutans* and *C. albicans* appear to colonize distinct dentinal tubules, suggesting that these microbes may behave differently in certain niches and instead of partners, they can become competitors (143). Ultimately, *S. mutans* manipulates the microbiome metabolism and composition to favor its prevalence in the oral cavity by forming partnerships with other residents of the microbiome to improve its fitness. Also, depending on the niche, *S. mutans* might perceive its previous partners as competitors and then continue solo.

## CLOSING REMARKS: CAN *S. MUTANS* RESEARCH STILL LEAD THE WAY?

From its identification roughly a century ago to the transformative investigations of the past 40–50 years that yielded major insights into bacterial genetics and physiology, and even overturned long-standing dogmas, *S. mutans* research has consistently driven progress in microbiology and is expected to continue to push the field forward. For example, efforts to elucidate how *S. mutans* senses and responds to environmental cues through interconnected regulatory networks that control stress tolerance and biofilm formation are likely to have broad implications, extending to other streptococci and closely related bacteria. In addition, the ready availability of fresh clinical samples and recent technological advances enabling 3D analysis of oral biofilms, including intact freshly obtained oral biofilms (123), have opened new perspectives on the biogeography of polymicrobial communities and on how specific and complex microbial interactions can steer the microbiome toward health or disease (123, 144–147). Finally, given its host-associated biofilm lifestyle, we expect that *S. mutans* will remain a powerful model organism for dissecting how bacteria coordinate metabolic processes, such as carbon flux, biofilm formation, and stress responses with virulence. Thus, the continued study of *S. mutans* is therefore essential not only for advancing our understanding of dental caries and informing the development of new therapeutic approaches to address this significant clinical problem but also for elucidating fundamental principles of bacterial physiology, environmental adaptation, and pathogenesis.
